# Nitrogen fixation by common beans in crop mixtures is influenced by growth rate of associated species

**DOI:** 10.1186/s12870-023-04204-z

**Published:** 2023-05-15

**Authors:** Akanksha Singh, Christian Schöb, Pietro P. M. Iannetta

**Affiliations:** 1 Agricultural Ecology Group, Institute of Agricultural Sciences, ETH Zurich, Zurich, Switzerland; 2 Department of International Cooperation, Research Institute of Organic Agriculture, Frick, Switzerland; 3 Área de Biodiversidad y Conservación, Universidad Rey Juan Carlos, 28933, Móstoles, Madrid, Spain; 4 Department of Ecological Sciences, The James Hutton Institute, Dundee, DD2 5DA Scotland, UK

**Keywords:** Biological nitrogen fixation, Common bean, Legumes, Crop mixtures, Plant nutrients

## Abstract

**Background:**

Legumes can fix atmospheric nitrogen (N) and facilitate N availability to their companion plants in crop mixtures. However, biological nitrogen fixation (BNF) of legumes in intercrops varies largely with the identity of the legume species. The aim of our study was to understand whether BNF and concentration of plant nutrients by common bean is influenced by the identity of the companion plant species in crop mixtures. In this greenhouse pot study, common beans were cultivated with another legume (chickpea) and a cereal (Sorghum). We compared BNF, crop biomass and nutrient assimilation of all plant species grown in monocultures with plants grown in crop mixtures.

**Results:**

We found beans to exhibit low levels of BNF, and to potentially compete with other species for available soil N in crop mixtures. The BNF of chickpeas however, was enhanced when grown in mixtures. Furthermore, biomass, phosphorous and potassium values of chickpea and Sorghum plants were higher in monocultures, compared to in mixtures with beans; suggesting competitive effects of beans on these plants. Concentration of calcium, magnesium and zinc in beans was higher when grown with chickpeas than with Sorghum.

**Conclusions:**

It is generally assumed that legumes benefit their companion plant species. Our study highlights the contrary and shows that the specific benefits of cereal-legume mixtures are dependent on the growth rate of the species concerned. We further highlight that the potential of legume-legume mixtures is currently undervalued and may play a strong role in increasing N use efficiency of intercrop-based systems.

## Background

Lack of nitrogen (N) is one of the main factors limiting crop productivity worldwide [1, 2]. Developing intercropping mixtures that include legumes is a promising approach for increasing N use efficiency in agricultural fields. Legumes fix atmospheric N by forming symbiotic associations with rhizobia, and it is estimated that legumes can fix more than 200 kg of N per hectare in both tropical and temperate systems [3–5]. Hence, legumes are not only less reliant on synthetic fertilizers for their N demand, but also, can provide additional N to the system in the form of green manure. However, the degree of biological nitrogen fixation (BNF) by legumes is strongly affected by their associated environmental conditions and varies amongst legume species [6, 7]. Due to such context dependency, legumes may not always benefit their associated species in intercropping systems and may even compete for resources. Such competitive interactions between legumes and other plants in mixtures have rarely been investigated.

The most widespread intercropping systems that incorporate legumes are legume-cereal mixtures, and these are still grown commonly in low-input systems in the tropics [8]. Facilitation and complementary use of resources are the two mechanisms that make legume-cereal mixtures productive [9]. As legumes and cereals differ in the way they find and use resources, there is resource use complementarity and limited interspecific competition [10, 11]. Legumes fix N and can also facilitate N availability to the cereal plants. However, niche differentiation or facilitative interactions between plants are dynamic and can turn into stronger competition if the biotic/abiotic environmental factors change [10, 12]. For example, studies investigating the effect of fertilizer application on legume-cereal mixtures show that such mixtures are usually suited for systems with low N availability [10, 11]. As the amount of available N increases, cereals generally benefit at the expense of the legume, potentially due to their faster growth rate and thereby accumulation of N [13–15]. Nodule formation in legumes, however, is a gradual process and initially legumes too are dependent on soil N [16]. Hence, in the early stages in crop mixtures there may be competition between a legume and a cereal for available N.

N-fixation is an energy intensive process and it is well established that legumes can preferentially absorb N in inorganic form (seed N and soil inorganic N) and fix atmospheric N only when there is low availability of N in the soil [17–19]. N-fixation is also dependent on several other factors such as soil pH or additional nutrients such as phosphorus [20, 21]*.* In crop mixtures one plant can mobilize otherwise unavailable nutrients for its companion plant. However, the type of nutrients mobilized is dependent on the functional trait of the plant species in the mixture [22]. For example, mobilization of soil phosphorous (P) and facilitation of its uptake has been observed in several cereal-legume mixtures [23–26]. The legume species has been identified as the causal agent mobilizing P in these mixtures and makes it available to its cereal companion plant. In contrast, mobilization of micronutrients such as zinc or iron in crop mixtures has been shown to be caused by cereal species [27, 28]. As N-fixation is affected by availability of other nutrients, the functional trait of the companion plant may further influence a legumes’ N-fixation ability.

In comparison with other legumes, common beans (*Phaseolus vulgaris* L.) are known to be poor N fixers [29, 30]. Nevertheless, beans are widely used in crop mixtures together with cereals. Similar to other legumes, N-fixation by beans is highly context dependent and can vary with several factors such as the bean variety or N availability in the soil [29, 31, 32]. Growing cereals with legumes was a traditional practice in temperate regions, although use of legumes was replaced in the twentieth century by synthetic fertilizers [3]. Lately beans are being promoted as a sustainable protein source and there is growing interest to develop intercropping systems including beans in temperate agricultural systems. Adoption of intercropping systems however often fails due to various economic, political and mechanistic barriers [33]. We argue that this further occurs because not all mixtures work well for the reasons described above. To develop productive crop mixtures, it is crucial to understand the mechanisms that promote complementarity in mixtures. In the context of beans, it is particularly important to determine factors that increase N-fixation potential or nutrient uptake because beans are poor N fixers.

This study is part of the DiverBeans project that aimed to increase bean production in North Macedonia using crop diversification measures. In a previous pot-based crop mixture experiment, we found that chickpeas (*Cicer arietinum* L.) and Sorghum (*Sorghum bicolor* (L.) Moench) significantly improved bean yield in comparison to bean monocultures. However, we observed that these effects were different for the different bean varieties [34]. In this study we have further explored the mechanisms that could explain the crop interactions we observed in our previous work.

The aim of our study was to understand whether N-fixation potential and concentration of essential plant nutrients by beans is influenced by the functional traits of the companion plant species in crop mixtures; and if these effects vary temporally or across the two bean varieties. Most studies that have explored N-fixation by legumes in mixtures have focused on cereal-legume mixtures while nutrient dynamics in legume-legume mixtures is largely ignored. Such mixtures hold vast potential for increasing N use efficiency in agricultural systems. We compared N-fixation, biomass and nutrient assimilation of bean plants in different cropping treatments (monocultures, bean variety mixtures and crop mixtures), across two different time points. The two companion plant species used were a cereal (Sorghum) and a legume (chickpea). We hypothesized that: (a) productivity of all species would be higher when grown in mixtures than when grown in monoculture; (b) beans would fix more N when grown with a cereal than when grown with another legume or in monoculture; and (c) concentration of N and P would be higher in beans when grown with chickpeas and the concentration of zinc would be higher when grown with Sorghum.

## Results

For all response variables we recorded similar effects for bean variety mixture and bean monoculture treatments. Therefore, for beans we only describe results related to monoculture and crop mixtures below.

### Biomass per plant for all species

**Fig. 1:**
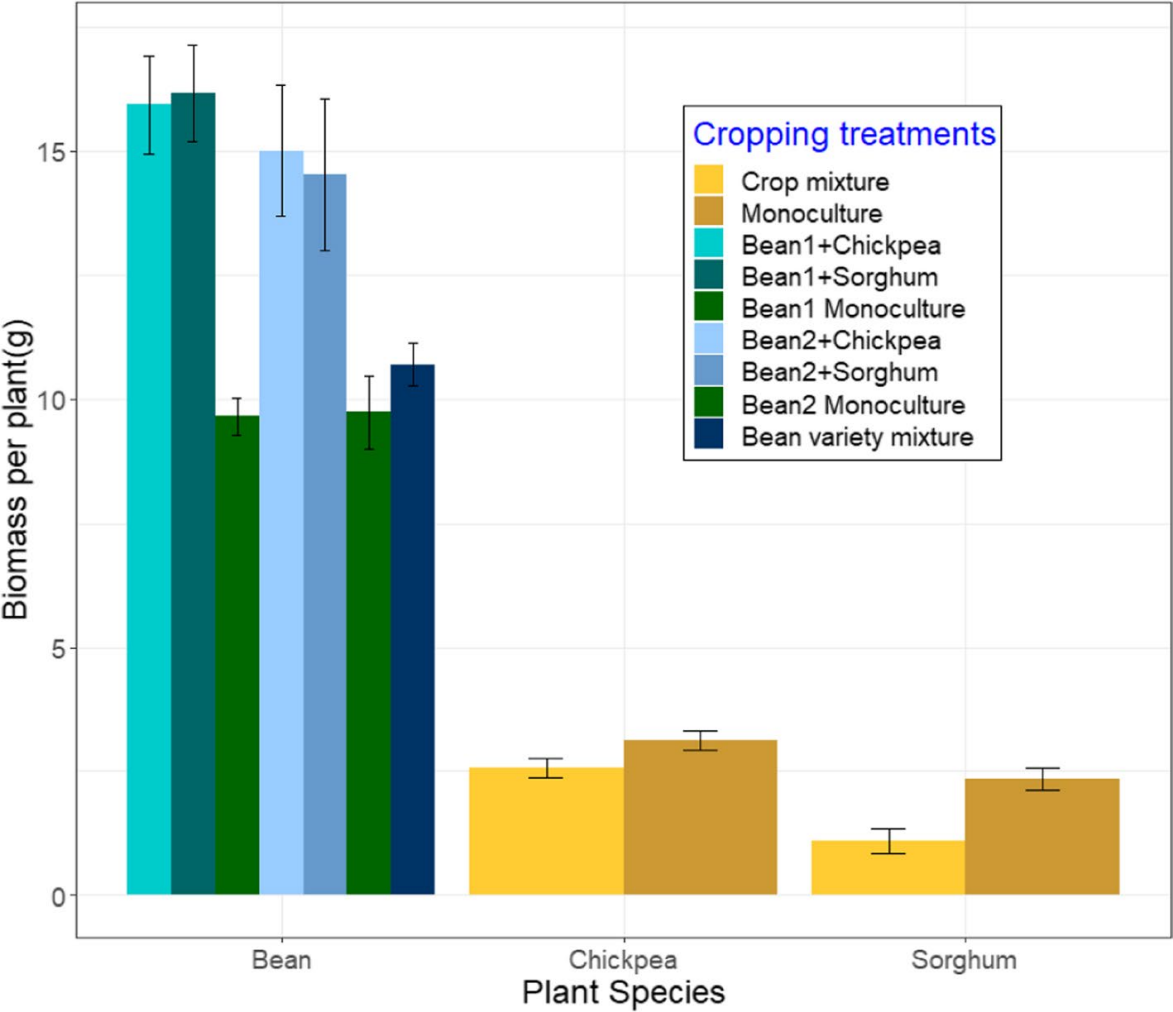
Biomass per plant of the different plant species in the different cropping treatments. (Week five and eight data are combined. Bean1 and bean2 refer to bean variety 1 and 2, respectively. Error bars represent ±1SE)

Biomass per plant of beans and Sorghum was affected by the cropping treatment (Table 1) but, the effect of this treatment varied for the two species. On average for the two varieties combined, bean biomass per plant was ~ 62.5% higher in crop mixtures than in monoculture, whereas, Sorghum (~ 53%) biomass was higher when grown in its monoculture (Fig. 1). Within the two crop mixtures, the identity of the companion crop species (i.e. Sorghum or chickpea) had no effect on bean biomass (*P* = 0.866). The two bean varieties also varied in their overall biomass per plant (Table 1), with variety 1 (13.4 ± 0.03) recording higher biomass than variety 2 (12.4 ± 0.04). When we analyzed the data separately for the 2 weeks, we recorded the bean varieties to vary in their biomass only in week five (F_1,69_ = 23.49, *P* < 0.001) and not in week eight (F_1,69_ = 0.241, *P* = 0.625). Biomass of variety 2 was lower than that of variety 1 in week five but, by week eight both varieties had similar biomass.

**Table 1:** Effect of explanatory variables on dry biomass, %ndfa and primary nutrient concentrations of the different plant species

		Response variables				
		Biomass/plant	%ndfa	%N	P	K
Explanatory variables	**Beans**					
	Cropping treatment	F_2,140_ = 46.88, *P* < 0.001	Week 8: F_2,68_ = 3.17, *P* = 0.049	–	–	F_2,121_ = 4.412, *P* = 0.014
	Bean variety	F = _1,140_ = 5.572, *P* = 0.021	–	–	F_1,121_ = 10.45, *P* = 0.001	F_1,121_ = 14.349, *P* < 0.001
	Week of collection	NA	NA	F_1,121_ = 291.66, *P* < 0.001	F_1,121_ = 52.89, *P* < 0.001	F_1,121_ = 17.959, *P* = 0.007
	Companion crop identity	–	Week 8: F_1,49_ = 4.89, *P* = 0.036	–	–	F_1,98_ = 7.48, *P* < 0.001
	**Chickpeas**					
	Cropping treatment	F_1,51_ = 3.21, *P* = 0.082	F_1,50_ = 5.01, *P* = 0.032	F_1,45_ = 26.47, *P* < 0.001	F_1,45_ = 14.601, *P* < 0.001	F_1,45_ = 1.051, *P* = 0.312
	Week of collection	NA	F_1,50_ = 6.10, *P* = 0.025	F_1,45_ = 139.26, *P* < 0.001	F_1,45_ = 57.348, *P* < 0.001	F_1,45_ = 29.049, *P* < 0.001
	**Sorghum**					
	Cropping treatment	F_1,51_ = 13.94, *P* < 0.001	NA	–	F_1,39_ = 35.823, *P* < 0.001	F_1,39_ = 21.643, *P* < 0.001
	Week of collection	NA	NA	F_1,39_ = 78.61, *P* < 0.001	–	F_1,39_ = 89.034, *P* < 0.001

Explanatory variables: cropping treatments (crop mixture, monoculture, variety mixture), week of collection (week 5 or 8) and bean variety (Variety 1 or 2). NA represents when a variable was not included in the model. ‘-‘sign represents when a variable was not significant

### N-fixation for beans and chickpeas

Bean plants did not fix nitrogen (N) by week five and they only started to fix by week eight. In week eight, cropping treatment and companion crop identity significantly affected BNF by bean plants (Table 1). Although, even by week eight BNF was low and positive in only: 61% of the pots (11 of the 18 pots) where beans were grown with Sorghum (mean 1.78 ± 0.37 mg of N fixed in the 11 pots), in two monoculture pots and in one variety mixture pot. Beans did not fix N in any pot when grown with chickpeas, and the two bean varieties did not differ in their BNF potential. There was a weak negative and significant correlation between total %N and %ndfa values for bean plants (t = − 1.993, df = 69, *P* = 0.050); suggesting a relationship between higher soil N uptake and reduced BNF capacity.

**Fig. 2:**
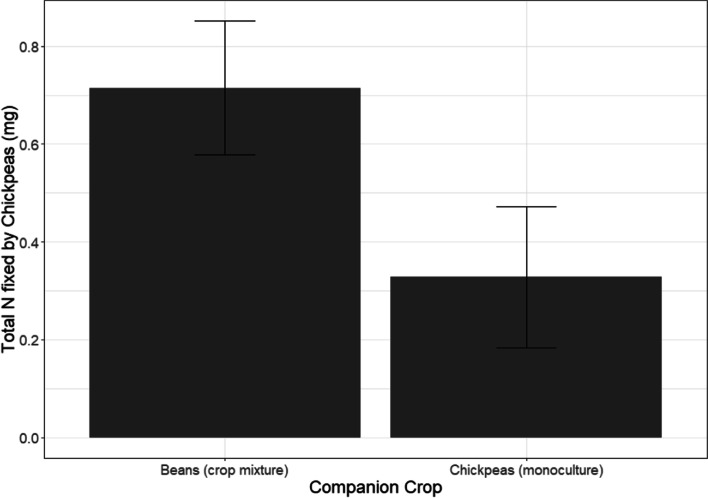
Total N fixed by chickpea plants in the cropping treatments (chickpea monoculture, chickpea with beans). *(Both harvest time points are combined. Error bars represent ± 1SE)*

Contrary to beans, chickpea plants started to fix N by week five and %ndfa of chickpea plants were higher in week five than in week eight (Table 1; week 5: 25.6%, week 8: 14.9%). Cropping treatment affected chickpea BNF (Table 1) and, chickpeas fixed more N when grown with beans than when grown in monoculture (Fig. 2). We recorded a weak negative correlation between chickpea ndfa values and biomass (t = − 2.433, df = 52, *P* = 0.018).

### Concentration of primary nutrients (NPK) in all plant species

**Fig. 3:**
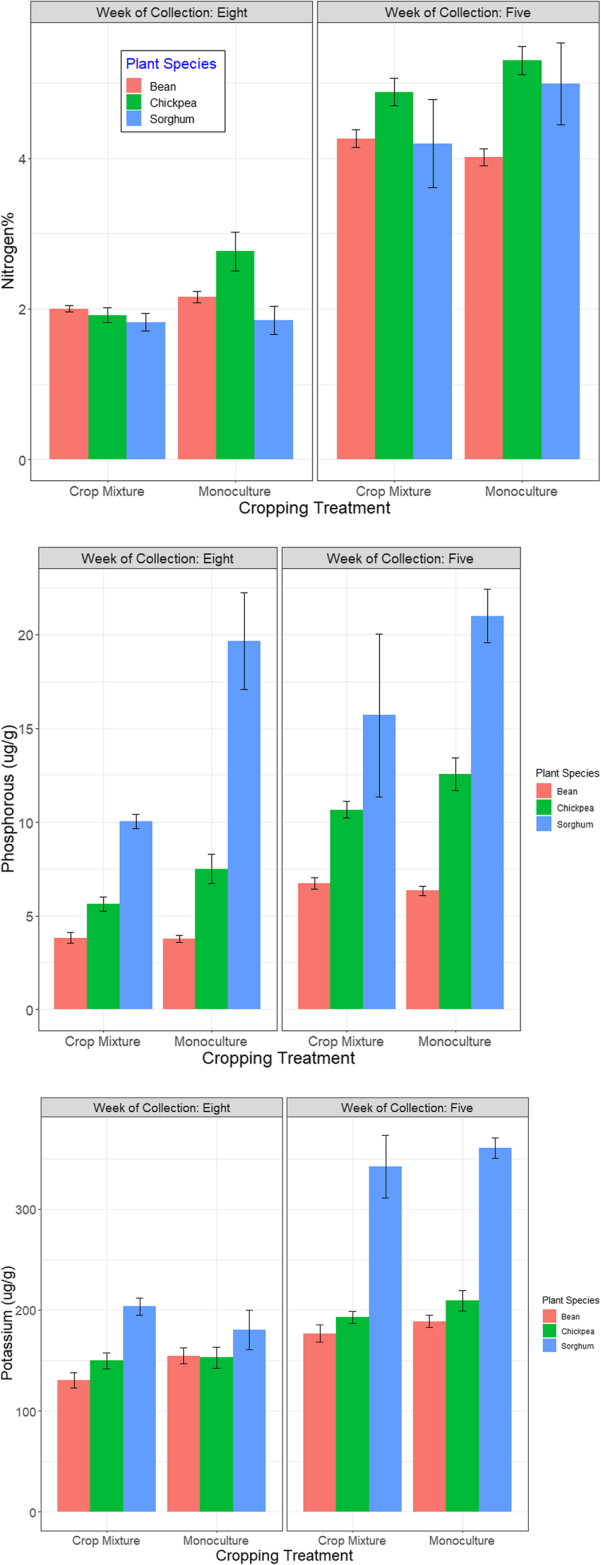
Variation in NPK concentrations in different plant species in the different cropping treatments. (Data of two harvest time points (week five and eight) is combined. Error bars represent ± 1SE)

For all species, concentrations of NPK were higher in week five than in week 8 (Table 1 and Fig. 3), only the P concentration of Sorghum did not vary across the two harvest time points (*P* = 0.17).

#### Bean

Combining both harvest time points together, the two varieties did not vary in their acquisition of %N (*P* > 0.05). Although, we did record variation in the %N of the two varieties in week five (F_1,68_ = 11.975, *P* < 0.05) and not for week eight (F_1,68_ = 0.993, *P* > 0.05); in week five higher %N was recorded in variety 2 (4.4 ± 0.02%) than in variety 1 (3.89 ± 0.18%). We observed no overall effect of cropping treatment on %N (*P* > 0.05; Fig. 3). However, in week eight we recorded a marginal effect of companion crop identity on %N (X^2^ = 0.134, *P* = 0.064); beans acquired higher %N when grown with Sorghum (2.11 ± 0.02%) than when grown with chickpeas (1.94 ± 0.009%).

Similarly, the two varieties varied in their concentration of P and K (Table 1); variety 2 had higher concentration of both elements compared to variety 1 (***Var1:****P* = 4.77 ± 0.03, K = 148 ± 0.72 μg/g; ***Var2:****P* = 5.56 ± 0.04, K = 177 ± 0.74 μg/g). Cropping treatment had no effect on P but, it did effect K concentration (Table 1, Fig. 3); K concentration was highest in bean plants when they were grown in their monocultures (Fig. 3). Within crop mixtures, we further recorded K concentration to be higher in beans when they were grown with chickpeas (143 ± 2.31 μg/g) than when grown with Sorghum (117 ± 2.41 μg/g) (Table 1).

#### Chickpea and sorghum

For chickpeas, K concentration did not vary across the two cropping treatments (*P* = 0.312) but we did observe P and N concentrations of chickpeas to be higher in their monoculture treatment (Table 1, Fig. 3). P and K concentrations of Sorghum in week five were higher in its monoculture treatment. In contrast, in week eight, Sorghum K concentration was higher in the crop mixture treatment, and its P concentration was recorded to be higher in the monoculture treatment (Table 1, Fig. 3). We recorded no significant effect of the cropping treatment on the %N concentration of Sorghum (*P* > 0.05).

### Concentration of secondary and micro-nutrients

Similar to NPK, we found concentrations of magnesium and zinc to be higher in week five than in week eight for all plant species (Tables 2 and 3). Calcium concentrations of only Sorghum plants were higher in week five (Tables 2 and 3). Manganese concentrations of both chickpea and bean plants were also higher in week five than in week eight (Tables 2 and 3).

**Table 2:** Effect of explanatory variables on the concentration of secondary and micro-nutrients in plant species

		Response Variables			
		Calcium	Magnesium	Manganese	Zinc
Explanatory variables	**Bean**				
	cropping treatment	Week 5: F = 4.34, *P* = 0.021	Week 8: F = 2.54, *P* = 0.098	Week 5: F = 3.92, *P* = 0.025	–
	Bean variety	–	Week 5: F = 16.93, *P* < 0.001	Week 5: F = 4.46, *P* = 0.043	Week 8: F = 4.25, *P* = 0.049
	Companion crop identity	Week 5: F = 3.58, *P* = 0.069	Week 5: F = 3.90, *P* = 0.0.058	–	Week 5: F = 3.06, *P* = 0.092
	Week of collection	–	F_1,121_ = 6.09, *P* = 0.029	F_1,121_ = 21.505, *P* = 0.008	F_1,121_ = 16.56, *P* = 0.001
	**Chickpeas**				
	cropping treatment	F_1,45_ = 4.284, *P* = 0.047	–	–	–
	Week of collection	–	F_1,45_ = 5.781, *P* = 0.02	F_1,45_ = 6.818, *P* = 0.019	F_1,45_ = 8.071, *P* = 0.007
	**Sorghum**				
	cropping treatment	–	–	–	–
	Week of collection	F_1,39_ = 17.849, *P* = 0.002	F_1,39_ = 14.648, *P* = 0.005	–	F_1,39_ = 11.533, *P* = 0.002

Explanatory variables: Cropping treatment (crop mixture, monoculture, variety mixture), week of collection (week 5 or 8) and bean variety (Variety 1 or 2). ‘-‘sign represents when a variable was not significant

**Table 3:** Concentration of secondary and micro-nutrients in the different harvest time points for all plant species

	Week of collection		Cropping treatment		Associated species	
	*Five*	*Eight*	*Crop Mixture*	*Monoculture*	*Chickpea*	*Sorghum*
**Beans**						
Calcium	–	–	Week 5: 104 ± 0.85	Week 5: 89.5 ± 0.87	Week 5: 110 ± 0.9	Week 5: 99 ± 1.21
Magnesium	12.39 ± 0.03	11.49 ± 0.08	Week 8: 10.34 ± 0.22	Week 8: 12.48 ± 0.24	Week 5: 13.13 ± 0.15	Week 5: 11.97 ± 0.11
Manganese	0.15	0.21	Week 5: 0.16	Week 5: 0.14	–	–
Zinc	0.12	0.08	–	–	Week 5: 0.14	Week 5: 0.08
**Chickpea**						
Calcium	–	–	Week 8: 57.9 ± 1.63	Week 8: 81.3 ± 0.73	NA	NA
Magnesium	14.83 ± 0.06	13.26 ± 0.16	–	–	NA	NA
Manganese	0.14	0.18	–	–	NA	NA
Zinc	0.19	0.11	–	–	NA	NA
**Sorghum**						
Calcium	15.2 ± 0.08	10.5 ± 0.12	–	–	NA	NA
Magnesium	14.91 ± 0.05	11.86 ± 0.08	–	–	NA	NA
Manganese	–	–	–	–	NA	NA
Zinc	0.19	0.12	–	–	NA	NA

All values are in ug/g and represent Mean ± SE. Only significant values are mentioned; i.e. when overall week/cropping treatment/associated species identity within a week had a significant effect

#### Beans

We found no effect of cropping treatment or bean variety on concentrations of the secondary and micro-nutrients in both harvests combined (*P* > 0.05). However, concentrations of calcium and manganese in week five and, marginally concentrations of magnesium in week eight, were all influenced by the cropping treatment (Tables 2 and 3). Concentrations of calcium and manganese was higher in crop mixtures than in monocultures (Table 3). Within the crop mixtures in week five, concentrations of calcium, magnesium and of zinc were higher in bean plants when grown with chickpeas (Tables 2 and 3). Even in week eight, we recorded higher concentration of calcium in bean plants in the presence of chickpeas (Tables 2 and 3). Variety 2 had significantly higher concentrations of magnesium and manganese in week five and of zinc in week eight, compared to variety 1 (Table 2).

#### Chickpeas and sorghum

Cropping treatment did not affect concentrations of any of the secondary and micro-nutrients for chickpea and Sorghum plants. The only exception was chickpea plant calcium concentration (Table 2); chickpea plants had significantly higher calcium concentration in their monocultures than when grown with beans in mixtures (Table 3).

## Discussion

Overall, our study shows that facilitative effects of legumes in crop mixtures are species dependent and are significantly influenced by traits such as growth rate of the interacting species. We further highlight the potential of legume-legume mixtures in increasing nutrient use efficiency in intercropping systems. We suggest that such context dependent effects of legumes on their companion species may occur because, being a biological process, BNF is highly sensitive to environmental conditions such as levels of synthetic N fertilization, levels of other soil nutrients, temperature, and rainfall/water availability etc. The mechanisms behind our results are discussed below.

Here we observed the biomass of beans to increase at the expense of Sorghum biomass. These results are contrary to other studies on legume-cereal mixtures which show a trade-off in the opposite direction i.e., the yield of an intercropped cereal increases, consequently reducing the yield of an intercropped legume, irrespective of available N [9, 10]. Plant-plant interactions are known to depend on changes in the environmental conditions or plant development [12, 35]. In our study the competitive effect of beans could have occurred due to their late nodule formation. Nodule formation and nodule activation in legumes is a gradual process [16, 36]. During the early growth period, legumes depend on soil mineral N and seed N reserves [7] and can be in direct competition for mineral N with their companion plants. We recorded no BNF activity in bean plants by week five. Hence, in the initial stage beans potentially competed with Sorghum for available soil N, depleting available N for Sorghum plants and consequently resulting in lower Sorghum biomass.

Another reason why beans did not form nodules or fix N by week five may have been that the concentration of soil N in our study was high. We did record a weak correlation between %N concentration of beans and their N-fixation rate (%ndfa). Biological nitrogen fixation (BNF) is an energy intensive process and legumes preferentially take up N in inorganic form than relying on BNF for their N demand [11]. We did not measure soil N concentration in our study but we did provide fertilizer to all our plants and, potentially the mineral N available in our potting soil was high. Studies have unanimously shown that as early availability of mineral N increases, cereals such as wheat or barley become more competitive and grow faster at the detriment of the legume [37]. Recent findings of Boudsocq et al. [38] show that such competitive effects of cereals over legumes can even occur with the increase in availability of mineral P. Although in our work we observed an opposite effect of high N availability, i.e., beans became more competitive and grew faster at the detriment of Sorghum. This suggests that competitive plant-plant interactions under high N availability are strongly influenced by the variation in growth rate of the interacting species.

Beans were the fastest growing species in our study and Sorghum is a slow growing cereal. Evidence of cereal-legume interactions have been derived from studies on faster growing cereal species such as barley, maize or wheat [37]. Interactions involving a slower growing cereal with a faster growing legume are understudied. During the initial growth phase, if a species grows faster than others, it would gradually dominate resource acquisition, compete for soil N and would greatly influence the growth and final performance of interacting species [13, 39, 40]. Such effects of growth rate in mediating plant interactions were evident in our study. Being the fastest growing species, beans had more access to light resources, assimilated highest concentration of nutrients and biomass; consequently, reducing biomass for the interacting species. Overall, late nodule formation, high soil nutrient concentration and fast growth rate of beans could explain why beans did not initially fix N and instead competed with other species for resources.

The potential high soil N concentration, however, did not affect the BNF ability of chickpeas. Chickpeas were recorded to fix N by week five. In fact, they fixed more N in week five than in week eight and fixed more N when grown with beans. N-fixation largely varies with the identity of the legume species [7]. Beans are known to be poor fixers relative to other species and this was evident in our study [41]. Reduction in BNF of chickpeas by week 8 could be explained by competition for carbon compounds with the reproductive organs that develop toward the end of the plant growth cycle [42, 43]. Nevertheless, our results suggest that legume-legume mixtures can increase N use efficiency in agricultural systems, in particular if a species with good N-fixation abilities (e.g. chickpea) is combined with fast growing poor N fixer (e.g. common beans).

Legume-legume mixture may further be efficient in the uptake of other nutrients because we recorded higher concentrations of potassium (K), calcium, and magnesium in beans in mixtures with chickpeas than in mixtures with Sorghum. Interestingly, chickpeas are known to mobilize phosphorous (P) acquisition for companion species [23] but we did not record P concentration to increase in beans with chickpea present. We hypothesize that this may have occurred because beans are also a legume. The mechanism by which legumes increase available P is by acidification of the rhizosphere and by the release of carboxylates and phosphatases [26, 38, 44]. In a legume-legume mixture as both species may have this mechanistic ability, P availability might be similar in a legume monoculture than in legume-legume mixtures. However, unlike other legumes, beans have not been shown to increase P uptake in their companion cereal species [45]. Hence, more mechanistic studies of the rhizosphere are needed to understand nutrient mobilization in legume-legume mixtures.

We had further hypothesized that Sorghum’s presence would facilitate uptake of zinc for bean plants. Instead, we recorded zinc concentrations in beans to be higher in the presence of chickpeas. Low growth and performance of Sorghum may explain this ‘no effect’. Irrespective of its growth though, we did record higher K concentrations in Sorghum for mixtures with beans than in its monocultures at week eight. K concentration of beans was higher in their monocultures. Increase in K uptake in a cereal when intercropped with a legume has been shown by previous studies [46] but the mechanisms underlying this effect have not been investigated. Our work suggests that facilitation of K instead of P could be one way in which beans can benefit cereal species. It is important to note though that this effect was only observed later in the growth cycle (week eight). For several other variables too, we found temporal variations regarding the effects of our experimental treatments. This suggests that measuring nutrient dynamics at different stages of plant growth would provide a better mechanistic understanding of facilitation in plant-plant interactions [40].

Studies that investigate competition or facilitation in crop mixtures usually base their conclusions on results obtained from one time point, usually at harvest. Species interactions however, vary over time and according to environmental conditions. For example, the two bean varieties in our study varied in their biomass and in their N concentration only at week five. If we had only collected data at this initial time point, we would have falsely concluded one variety to perform better than the other. Instead, we recorded the slower growing bean variety to overall assimilate higher concentrations of P and K than the faster growing bean variety. Hence, a slower growing variety potentially has a higher nutrient profile, making it more valuable for human consumption. Similar to these initial temporal variation for varieties, we recorded intercropping treatment to have an effect on magnesium and calcium concentration of bean plants only in week five; concentrations of these elements in bean plants were higher in crop mixtures in week five. Understanding the time point at which facilitation occurs between interacting plant species can be useful for proposing crop management measures in crop mixtures.

## Conclusions

Intercropping systems have numerous advantages but performance of intercrops is greatly influenced by factors such as plant species involved, plant density, plant varieties and available nutrients. Hence, generalization of optimal combinations is difficult and it is important to understand the many different mechanisms that reduce competition between interacting species. As our study was a pot study, we can only suggest the potential of the mechanisms highlighted in our study. It is important to test our results in the field under varying soil, fertilizer or plant density conditions. However, as there are multitude of mechanisms and production objectives involved in designing high yielding crop mixtures, experimental studies can only provide limited insights. Therefore, we further suggest that modelling of diverse cropping systems under a variety of scenarios is needed for better adaption of intercrops to local contexts. Such work would be crucial to enhance BNF by legumes and to further increase nutrient use efficiency in intercropping systems.

## Methods

### Study species

We used two bean varieties (Barlotto ‘Sasso Rosso’ and Barlotto ‘Taylors’) and two crop species (chickpeas and Sorghum). The bean varieties were purchased from Bioseme (Italy). The chickpea variety used in our experiment is a local unnamed Macedonian variety. The Sorghum variety used is called ‘Quartett’ and was purchased from Sativa Rheinau (Switzerland). The corresponding author of the manuscript undertook the formal identification of the plant material used in the study. As this is commercially available plant material (for agriculture use), a specimen of this material is not deposited in a publicly available herbarium.

### Study design

The two bean varieties Sasso Rosso and Taylors’, as well as Sorghum, and chickpea plants were grown as monocultures in pots. The bean varieties were also grown in variety mixtures and with the other two companion plant species, resulting in nine crop combinations in total (Bean Variety 1 monocrop, Bean Variety 2 monocrop, Variety 1 + Variety 2, Variety 1 + Sorghum, Variety 1 + chickpea, Variety 2 + Sorghum, Variety 2 + chickpea, chickpea monocrop, Sorghum monocrop). Each of these combinations were repeated 18 times. Our pots were divided into 18 blocks and each of the blocks contained one replicate of all the nine crop combinations. Within a block, pots with different crop combinations were placed randomly.

To estimate the temporal variation in BNF and in the concentration of nutrients, plant material was collected two times: on week 5 after sowing (beginning of flower formation) and on week 8 after sowing (fruit pod formation and filling). We used the ^15^N natural abundance method to estimate N-fixation efficiency [41]. The method is destructive and whole aboveground shoot material was harvested during each data-collection week from nine replicates of each of the crop combinations.

We further grew the two bean varieties separately in pots to estimate their ‘B’ value (more details under section ‘nitrogen fixation method’). We grew each of the two varieties in 20 pots; half of the pots for each variety were inoculated with rhizobia and half were not. Plant material was collected from 10 pots per variety (5 inoculated and 5 non-inoculated) during each data collection time point, i.e. week five and eight, for estimation of the ‘B’ value.

### Study set-up

We used 5 L pots of 18 cm length and 18 cm breadth and these were filled with the potting substrate or with sand+perlite (for the ‘B’ value plants). The potting substrate used is called Rasenerde 146 (Ricoter, Switzerland), has a 7.3 pH and the following composition: sand (30%), sterilized topsoil (30%), coco-peat (20%), and perlite (20%).

Bean seeds were surface sterilized with sodium hypochlorite 10% solution for 15 minutes. After this, we washed the seeds with distilled water. Then we coated the seeds with the inoculant mixture before sowing it in the potting substrate or in the sand+perlite mixture. Plant density in all pots was constant and we grew two plants per pot. Therefore, in monoculture pots we grew two plants of the same variety; in variety mixture pots we grew one plant of each of the two varieties and in crop mixture pots we grew one bean plant with another companion plant species.

One week after sowing we added 1 g of fertilizer (~ 3.09 kg per ha, Biosol Universaldünger vegan, Andermatt Biogarten) to all pots except for ‘B’ value pots, which contains 6% N, 0.5% P and 0.3% K. Three weeks after sowing we again added 1.5 mL of the bacterial inoculant solution to each bean plant. The inoculant used in the experiment was prepared from *Rhizobium tropici* 11418 strain (purchased from DSMZ, German Collection of Microorganisms and Cell Cultures GmbH, Leibniz Institute).

Data collection occurred on the 5th and 8th week after sowing. We collected plants of all species from monoculture and from crop mixture pots. The entire plant shoot was collected in paper bags from nine replicate pots from all crop combinations. These plants were then dried at 60 °C for 4 days, their dry biomass measured and milled to a fine powder for further analysis. The plants were then analyzed for their ^15^N, total N, and other macro and micronutrients (Phosphorous, Calcium, Potassium, Magnesium, Manganese and Zinc).

Potting medium for the ‘B’ value plants consisted of 50% sand and 50% perlite. Both the pots and the potting media were autoclaved and sterilized before use. The ‘B’ value plants were given a McKnights’ N-free nutrient solution [41] twice a week, and so were solely dependent on symbiotic N-fixation for N.

Data for ‘B’ value plants was collected at the same time as for other experimental plants (i.e. week 5 and 8), and an entire plant shoot was collected from five replicate pots per variety. We also collected nodules from the ‘B’ value plants in week 8 to estimate the identity of the colonizing bacteria. The PCR test revealed living colonies of *Rhizobium tropici* in the nodules of the ‘B’ value inoculated bean plants but not in the nodules of the non-inoculated plants.

All plants were watered on average with ~ 350 ml distilled water per week throughout the experiment.

The experiment was carried out at Lindau experimental station of ETH Zurich in Eschikon, Switzerland. Greenhouse temperature was maintained at (max:min) 20:16 °C, average humidity was 50.1%, and we used 16/8 h (Light/Dark) light cycle throughout the experiment. The photosynthetic photon flux density was approximately 338 μmol/m^2^/s and the light source was from cool-white fluorescent lamps.

### N/^15^N measurement

Test samples of each legume and bean variety were analyzed for N and ^15^N, once with 2 mg and once with 4 mg weight per tin capsule, to determine which amount of plant material gives the most reliant indication of N. The test samples indicated that between 2 and 2.2 mg per sample gives the best results. For each sample, a tin capsule was filled with 2 to 2.2 mg of either bean, Sorghum, or chickpea samples. The samples were then measured for their ^15^N as well as their N ratio with the isotope ratio mass spectrometer (IRMS) at the stable isotope lab of ETH Zurich. With the obtained δ^15^N results we calculated the %Ndfa through the ^15^N natural abundance method calculation.

### Determining concentration of additional nutrients

Plant nutrients were analyzed using the inductively coupled plasma – mass spectroscopy (ICP-MS) technique conducted at the inorganic environmental geochemistry lab of ETH Zurich. To prepare the samples we first measured 200 mg of individual samples in digestion tubes. Then samples were digested using nitric acid and hydrogen peroxide at 120 °C in the digestion chamber. The digested samples were then put in the ICP-MS (Agilent 7900, Agilent Technologies, United States) to determine their nutrient concentrations.

### Nitrogen fixation (N-fixation) method

We have used the ^15^N natural abundance method to estimate N-fixation efficiency in our experiment [41]. Reference plant (Sorghum grown in monocultures) and ^15^N values of beans and chickpeas were expressed in delta units (δ) which is a measure of the ^15^N content of the sample in parts per thousand (‰) relative to atmospheric N_2_: (sample atom% ^15^N minus 0.3663)/(0.3663) × 1000 [47]. These “delta units” were then used to calculate %Ndfa using the equation below:

$$\% Ndfa=\frac{\updelta\, {}_{15}\textrm{N}\ \textrm{of}\ \textrm{reference}\ \textrm{plant}-\updelta\, {}_{15}\textrm{N}\ \textrm{of}\ \textrm{N}_{2}\ \textrm{fixing}\ \textrm{legume}}{\updelta\, {}_{15}\textrm{N}\ \textrm{of}\ \textrm{reference}\ \textrm{plant}-\textrm{B}}\ast \frac{100}{1}$$

where ‘B’ for beans is the ^15^N value of shoots of legumes (beans or chickpeas) that are fully dependent upon N_2_ fixation and sampled at the same growth stage as the other experimental plants. For estimation of this ‘B’ value, the bean plants were grown in a N free sand media. Since the ‘B’ value was not assessed for chickpeas during the experiment, an estimated ‘B’ value of − 1.65 was used [48].

### Data analysis

We ran linear and generalized linear mixed effect models (LMER and GLMER) using the lmer package. To account for spatial variation, block was included as a random effect in all our mixed effect models. We also tested for interactions between the fixed effects in all our models. The data were analyzed using R version 4.0.2 in RStudio version 1.3.1056.

For all plants we analyzed data for the following response variables: biomass per plant, % nitrogen (N), and concentrations of phosphorous (P), potassium (K), calcium (Ca), Magnesium (Mg), Manganese (Mn) and zinc (Zn). For beans and chickpeas, we further analyzed data for the proportion of N derived from air (by BNF) (%ndfa). GLMER models with “binomial” family of error distribution and ‘pot number’ as a weighted factor were used to analyze %N data, for both harvest time points combined. When we analyzed the %N variable for a respective time point (week 5 or 8), we used GLM model with ‘quasibinomial’ family of error distribution because ‘Block’ as a random effect had zero variation in the GLMER models. For all other variables we used LMER models.

#### Beans

For all response variables (except %ndfa), we first ran a LMER or GLMER model on the whole bean data, including cropping treatment [crop mixture /bean variety mixture/bean monoculture] and week of collection (week five and eight) as fixed effects. To test for the effects of variety identity and identity of companion plant species on our response variables, we split the data and focused on single variety pots (i.e., bean monoculture and crop mixture pots). We included variety identity (Variety 1 or Variety 2), presence/absence of companion plant species and week of collection as fixed effects. For %ndfa response variable, only data for week eight was used.

Week of collection had a significant effect on all other response variables. Therefore, we further split the data for the different weeks and tested the effects of cropping treatment, bean variety identity and companion crop identity within each harvest time point for our response variables.

#### Chickpeas and sorghum

For all response variables for a specific plant, we only used data where the specific species was present. We first ran a LMER or GLMER model on the whole chickpea or Sorghum data, including cropping treatment [crop mixture and chickpea/Sorghum monoculture] and week of collection (week five and eight) as fixed effects. We further split the data into each harvest time point to investigate the effect of cropping treatment on all response variables.

## Data Availability

The datasets used during the current study are available from the corresponding author on reasonable request. This raw data supporting the conclusions of the manuscript will be uploaded on Zenodo after the publication of the manuscript.

## Abbreviations

*BNF*: Biological nitrogen fixation
*%ndfa*: Proportion of nitrogen derived from air by biological nitrogen fixation
*N*: Nitrogen
*P*: Phosphorous
*K*: Potassium
*Mg*: Magnesium
*Ca*: Calcium
*Mn*: Manganese
*Zn*: Zinc
*SE*: Standard error
*LMER*: Linear mixed effect models
*GLMER*: Generalised linear mixed effect models
*ICP-MS*: Inductively coupled plasma – mass spectroscopy
*IRMS*: Isotope ratio mass spectrometer
